# The use of prophylactic fluconazole in immunocompetent high-risk surgical patients: a meta-analysis

**DOI:** 10.1186/cc3883

**Published:** 2005-10-25

**Authors:** Kwok M Ho, Jeffrey Lipman, Geoffrey J Dobb, Steven AR Webb

**Affiliations:** 1 Consultant Intensivist, Department of Intensive Care, Royal Perth Hospital, Australia; 2 Professor and Head of the Department, Department of Intensive Care Medicine, Royal Brisbane Hospital, University of Queensland, Australia; 3 Acting Head of the Department, Department of Intensive Care, Royal Perth Hospital, Australia and Associate Professor, School of Medicine and Pharmacology, University of Western Australia, Australia; 4 Consultant Intensivist, Department of Intensive Care, Royal Perth Hospital, Australia and Senior Lecturer, School of Medicine and Pharmacology, University of Western Australia, Australia

## Abstract

**Introduction:**

High-risk surgical patients are at increased risk of fungal infections and candidaemia. Evidence from observational and small randomised controlled studies suggests that prophylactic fluconazole may be effective in reducing fungal infection and mortality. We evaluated the effects of prophylactic fluconazole on the incidence of candidaemia and hospital mortality in immunocompetent high-risk surgical patients.

**Methods:**

Randomised controlled studies involving the use of fluconazole in immunocompetent high-risk surgical patients from the Cochrane Controlled Trial Register (2005, issue 1) and from the EMBASE and MEDLINE databases (1966–30 April 2005), without any language restriction, were included. Two reviewers reviewed the quality of the studies and performed data extraction independently.

**Results:**

Seven randomised controlled studies with a total of 814 immunocompetent high-risk surgical patients were considered. The use of prophylactic fluconazole was associated with a reduction in the proportion of patients with candidaemia (relative risk [RR] = 0.21, 95% confidence interval [CI] = 0.06–0.72, *P *= 0.01; *I*^2 ^= 0%) and fungal infections other than lower urinary tract infection (RR = 0.39, 95% CI = 0.24–0.65, *P *= 0.0003; *I*^2 ^= 0%), but was associated with only a trend towards a reduction in hospital mortality (RR = 0.82, 95% CI = 0.62–1.08, *P *= 0.15; *I*^2 ^= 7%). The proportion of patients requiring systemic amphotericin B as a rescue therapy for systemic fungal infection was lower after prophylactic use of fluconazole (RR = 0.35, 95% CI = 0.17–0.72, *P *= 0.004; *I*^2 ^= 0%). The proportion of patients colonised with or infected with fluconazole-resistant fungi was not significantly different between the fluconazole group and the placebo group (RR = 0.66, 95% CI = 0.22–1.96, *P *= 0.46; *I*^2 ^= 0%).

**Conclusion:**

The use of prophylactic fluconazole in immunocompetent high-risk surgical patients is associated with a reduced incidence of candidaemia but with only a trend towards a reduction in hospital mortality.

## Introduction

Fungi are an increasingly important cause of nosocomial infections in intensive care units (ICUs) [1,2]. Systemic fungal infections are difficult to diagnose and are associated with substantial morbidity, attributable mortality, prolonged hospital stay, and healthcare costs [1-6]. Despite advances in medical technology and the development of new antifungal drugs, the crude and attributable mortality of candidaemia has remained unchanged in the past 20 years [7]. *Candida *spp. remain the commonest type of fungal infections in the ICUs and candidaemia accounts for 15% of all nosocomial bloodstream infections in the United States [1], with similar trends being reported worldwide [8].

The use of prophylactic antifungal therapy in ICU is controversial, although evidence from observational studies suggests that antifungal prophylaxis is associated with a reduced risk of candidaemia [9]. The risk factors associated with candidaemia are prevalent in high-risk or critically ill surgical patients, and these include the presence of a central venous catheter, acute renal failure, total parenteral nutrition, gastrointestinal perforation, and prior surgery [9,10]. Antifungal prophylaxis appears more beneficial for non-neutropenic critically ill surgical patients than for critically ill medical patients [10,11].

An antifungal agent selected for prophylaxis should have an appropriate spectrum of activity, should be easily delivered, and should have few adverse events [11]. Fluconazole appears suitable and its efficacy has been evaluated in several randomised controlled clinical trials involving high-risk surgical patients, with variable results. In addition to its antifungal activity, fluconazole has been demonstrated to bind to neutrophil surface receptors and to upregulate intracellular signalling pathways, leading to enhanced oxygen free radical release and chemotaxis *in vitro *[12]. It has been postulated that this immunomodulation effect may explain, at least in part, the beneficial effect of fluconazole on clinical outcome in patients with gut perforation [13]. We conducted a meta-analysis to investigate the effects of prophylactic fluconazole on the incidence of candidaemia and hospital mortality in immunocompetent high-risk surgical patients.

## Materials and methods

The literature search was performed on the Cochrane Controlled Trials Register (2005, issue 1) and the EMBASE and MEDLINE databases (1966–30 April 2005). Only randomised control clinical trials involving immunocompetent critically ill or high-risk surgical adult patients were included. For studies involving a mixture of surgical and non-surgical patients, only data from the surgical subgroup of patients were retrieved if possible. Studies involving the use of fluconazole antifungal prophylaxis for liver transplantation or for neutropenic cancer patients were excluded because they included immunosuppressed patients.

During the electronic database search, the following exploded MeSH terms were used: 'fluconazole' or 'antifungal' with 'critically ill', 'intensive care', 'trauma' or 'burns'. The reference lists of related reviews and identified original articles were searched for relevant trials. Finally, to ensure all suitable studies were included, the websites of the International Network of Agencies of Health Technology Assessment and the International Society of Technology Assessment in Health Care were searched and the company manufacturing fluconazole (Medical Department, Pfizer Australia Pty. Ltd., West Ryde NSW 2114, Australia) was contacted. If necessary, the authors of the identified trials were contacted to obtain additional information and unpublished data that were important in the analysis. No studies published in languages other than English were found in the literature search.

Two independent reviewers examined the titles and the abstracts of all identified trials to confirm they fulfilled the inclusion criteria. They examined and recorded the trial characteristics and outcomes independently, using a predesigned data abstraction form. This abstraction form was used to record information regarding the quality of the trial such as allocation concealment, the randomisation method, blinding of treatment, and the inclusion and exclusion criteria. The grading of allocation concealment was based on the Cochrane approach (i.e. adequate or uncertain or clearly inadequate). Any disagreements between the two independent reviewers were resolved by consensus. Any duplicated publications were combined to represent one single trial. Data were checked and entered into the Review Manager (version 4.2.6 for Windows, 2003; The Cochrane Collaboration, Oxford, UK) database for further analyses.

The hospital mortality and the proportion of patients with candidaemia were chosen as the main outcomes of this meta-analysis because they are the most specific clinically relevant outcomes of invasive fungal infections. There were no missing data for these two main outcomes in the included studies. The other outcomes assessed in this study included the proportion of patients colonised with or infected with fluconazole-resistant fungi, the proportion of patients requiring rescue therapy by systemic amphotericin B treatment, the proportion of patients with an adverse effect requiring cessation of the study drug, the proportion of patients with fungal infections other than urinary tract infection, and the total length of hospital stay. Urinary fungal infection is difficult to distinguish from colonisation, and for this reason these infections were excluded from further analyses in the present study. The definition of prophylaxis failure requiring amphotericin B treatment varied between different studies, but the common definition involved clinical deterioration with positive fungal culture from blood, deep tissue, or sputum.

### Statistical analyses

The differences in categorical outcomes between the treatment group and the placebo group were reported as the relative risk (RR) with the 95% confidence interval (CI), using a random effect model. The difference in the total length of hospital stay between the fluconazole group and the placebo group was reported as weight mean difference in days, using a random effect model. The presence of heterogeneity between trials was assessed by chi-square statistics and the extent of inconsistency was assessed by *I*^2 ^statistics [14]. Sensitivity analyses were conducted after excluding one study with unclear allocation concealment and one study that recruited some medical patients in the trial. The publication bias was assessed by funnel plot using hospital mortality as an endpoint.

## Results

**Figure 1:**
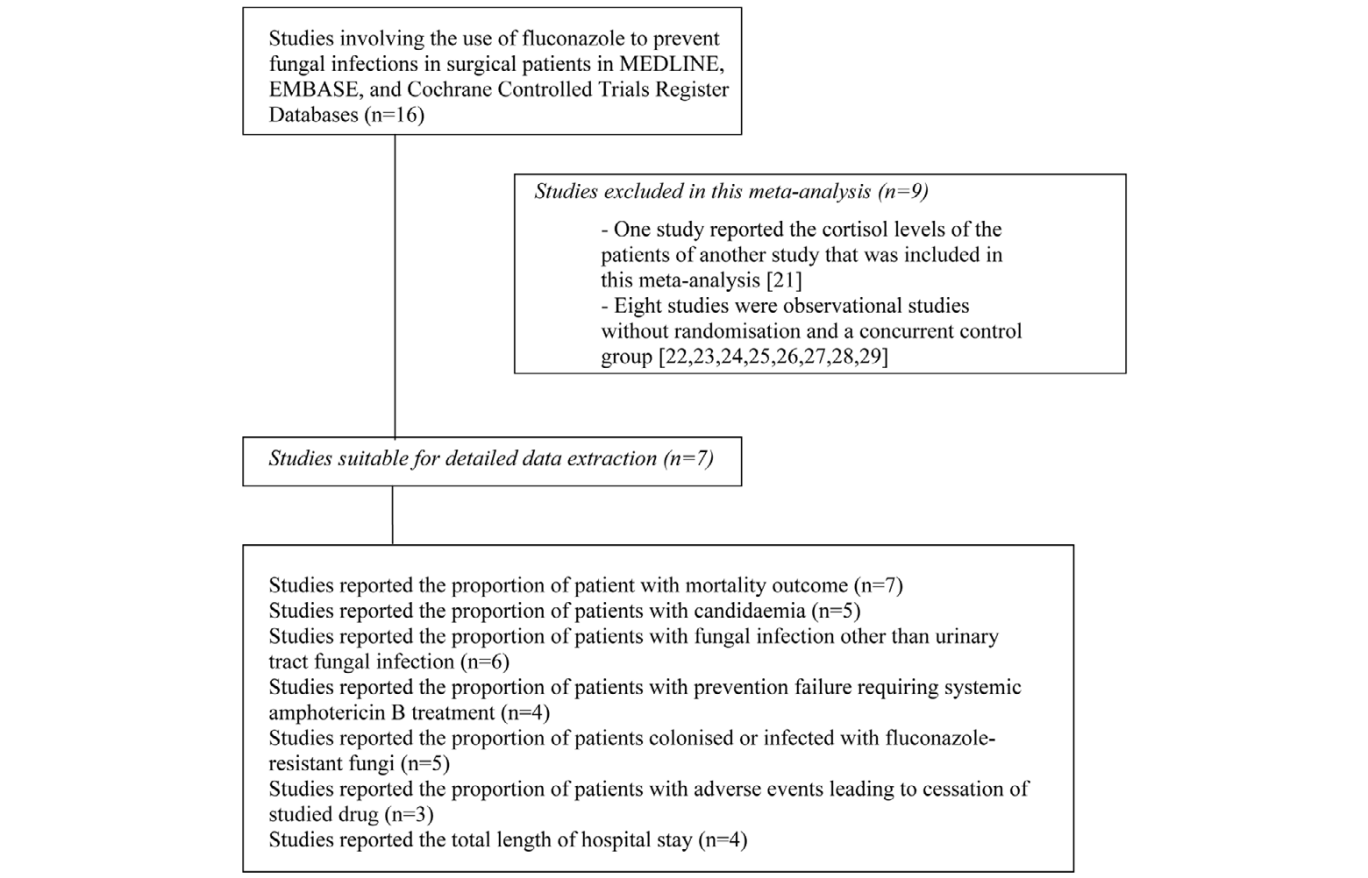
Flow chart showing study inclusion and exclusion in this meta-analysis.

We identified 16 potentially eligible studies, of which seven studies [13,15-20] fulfilled the inclusion criteria and were subject to meta-analysis (Figure 1). Five studies used the intravenous route [13,15,17-19], one study used the enteral route [16], and one study used either the intravenous or enteral route to administer the study drug depending on the function of the gastrointestinal tract [20]. The doses of fluconazole ranged from 100 to 800 mg/day. One study used a single intraoperative dose of fluconazole [18], and the other six studies used a prolonged course of prophylaxis until recovery from the surgical illness or until a new onset of symptoms or until a positive culture of fungi with the clinical diagnosis of invasive fungal infection. One study recruited patients with acute pancreatitis [19], one study recruited patients with septic shock secondary to intra-abdominal sepsis [13], two studies recruited patients with gut perforation [17,18], and three studies recruited general surgical and trauma patients [15,16,20]. The mean Acute Physiology and Chronic Health Evaluation II and Acute Physiology and Chronic Health Evaluation III scores ranged from 18 to 19 and from 63 to 65, respectively. Six studies had adequate allocation concealment and were definitely double-blinded. The details of all included studies are described in Table 1.

**Figure 2:**
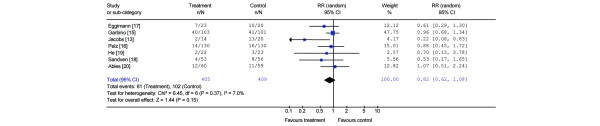
Forest plot showing the effect of prophylactic fluconazole on hospital mortality. RR, relative risk; CI, confidence interval.

**Figure 3:**
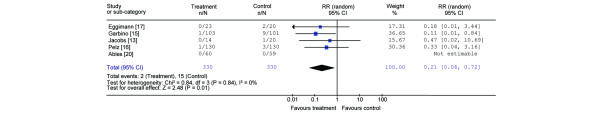
Forest plot showing the effect of prophylactic fluconazole on the proportion of patients with candidaemia. RR, relative risk; CI, confidence interval.

**Figure 4:**
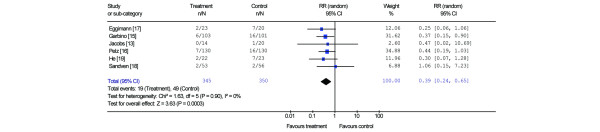
Forest plot showing the effect of prophylactic fluconazole on the proportion of patients with fungal infections. RR, relative risk; CI, confidence interval.

**Figure 5:**
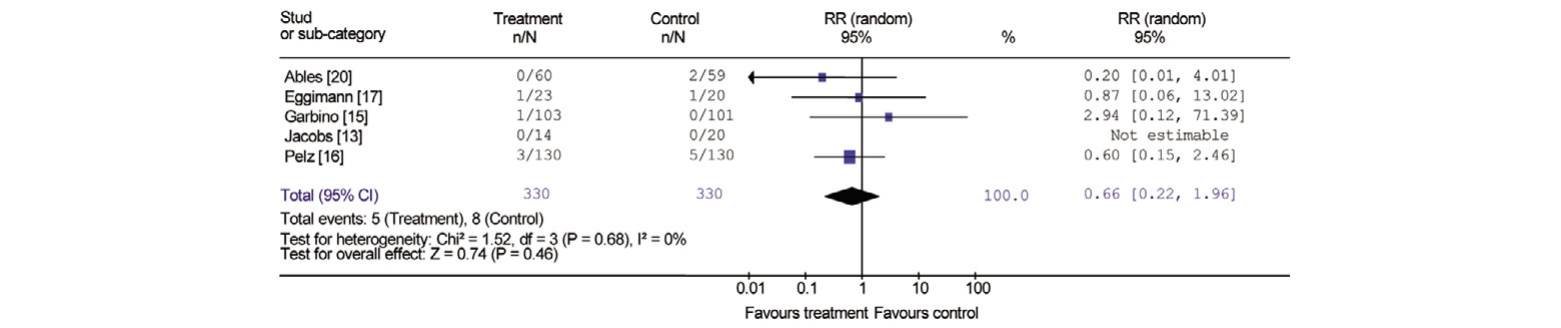
Forest plot showing the effect of fluconazole on proportion of patients colonised with fluconazole-resistant fungi. RR, relative risk; CI, confidence interval.

There was a good overall consistency in the results, without significant heterogeneity. The use of prophylactic fluconazole was associated with a reduction in the proportion of patients with candidaemia (RR = 0.21, 95% CI = 0.06–0.72, *P *= 0.01; *I*^2 ^= 0%) and fungal infections other than lower urinary tract infection (RR = 0.39, 95% CI = 0.24–0.65, *P *= 0.0003; *I*^2 ^= 0%), but was associated with no significant difference in hospital mortality (RR = 0.82, 95% CI = 0.62–1.08, *P *= 0.15; *I*^2 ^= 7%) (Figures 2, 3, 4). The proportion of patients requiring systemic amphotericin B as a rescue therapy for systemic fungal infection was lower after prophylactic use of fluconazole (RR = 0.35, 95% CI = 0.17–0.72, *P *= 0.004; *I*^2 ^= 0%). The proportion of patients colonised with or infected with fluconazole-resistant fungi (RR = 0.66, 95% CI = 0.22–1.96, *P *= 0.46; *I*^2 ^= 0%) (Figure 5) and the proportion of patients with adverse events leading to cessation of the study drug (RR = 0.75, 95% CI = 0.22–2.58, *P *= 0.65; *I*^2 ^= 0%) were not different between the fluconazole group and the placebo group. The total length of hospital stay was no different between the fluconazole group and the placebo group (weight mean difference = -0.4 days, 95% CI = -10.35 to 9.54, *P *= 0.94; *I*^2 ^= 52.4%).

Excluding one study with unclear allocation concealment [19] and one study that recruited some medical patients [15] did not affect the magnitude and significance of the results. None of the studies included a formal cost-effectiveness analysis. Five studies received financial grant or drug support from Pfizer Pharmaceuticals, Inc. – of which three studies stated explicitly that the funding agency was not involved in the collection and analyses of the data.

## Discussion

### Significance of our findings

This meta-analysis shows the benefits of fluconazole prophylaxis on most of the clinically relevant outcomes in critically ill or high-risk surgical patients. Fluconazole prophylaxis is associated with a much lower risk of candidaemia (RR = 0.2) and other candidal infections (RR = 0.4), with less requirement for systemic amphotericin B as a rescue therapy, and with a very safe adverse event profile, and is not associated with a significant increase in fluconazole-resistant fungi. However, we could only observe a trend towards a modest reduction in hospital mortality.

The candidaemia rate of 4.5% in the placebo arm of this meta-analysis is consistent with the estimated risk of candidaemia in patients with at least one risk factor for candidaemia. The risk factors include total parenteral nutrition, acute renal failure, central venous catheter, broad-spectrum antibiotics, immunosuppression, and prior surgery [9]. The beneficial effect of fluconazole on the risk of candidaemia as demonstrated in this meta-analysis is also consistent with the results of a cohort study on critically ill surgical patients [9] and with the results of fluconazole prophylaxis in immunosuppressed patients [30,31]. If our results are valid, the beneficial effect of fluconazole on candidaemia will be stronger than the effect of prophylactic topical non-absorbable antifungal agents such as amphotericin B or nystatin in critically ill patients [32].

Although prophylactic fluconazole is effective in reducing candidaemia, our results did not demonstrate a statistical significant reduction in hospital mortality. However, the candidaemia rate in the placebo arm of this meta-analysis is 4.5% and the expected absolute risk reduction in hospital mortality is about 2.25% if we assume that the attributable mortality of candidaemia is 50% [7]. If the 20% RR reduction in hospital mortality as demonstrated in this meta-analysis is valid, a prospective randomised controlled trial (or a meta-analysis) of more than 2,000 patients will be needed to demonstrate such a reduction in hospital mortality, assuming that the baseline hospital mortality of the study population is 25%. The number of patients considered in this meta-analysis was therefore too small to evaluate a mortality difference. The expected smaller treatment effect of prophylactic fluconazole on hospital mortality compared with candidaemia also suggests there are other important factors in determining mortality in patients at high risk of invasive fungal infections [33], and any potential beneficial immunomodulation effect of fluconazole, as suggested by some authors [12,13], is unlikely to be clinically significant in addition to its antifungal activity.

A pharmacoeconomic or cost-effectiveness analysis was not performed in the studies included in this meta-analysis. Based on the baseline risk of candidaemia of 4.5% in the placebo arm of this meta-analysis, the number of patients needed to treat is about 25 to prevent one episode of candidaemia. The cost of 200 mg fluconazole is about US$35 per day in Australia and Switzerland [17] and a 2-week prophylactic course of fluconazole will therefore cost US$490 per patient. The cost to prevent one documented episode of candidaemia is estimated to be US$12,250, which is equivalent to 40% of the economic cost of an episode of candidaemia (US$30,000) [34]. Prophylactic fluconazole may therefore be potentially cost-effective and justified in some high-risk surgical patients if the candidaemia rate in the selected ICU is high despite optimising other preventive measures such as vigorous hand hygiene, central venous catheter care, and prudent antimicrobial use [7].

Emergence of resistant fungi with widespread use of a prophylactic antifungal agent is a concern even if the drug is cost-effective. Our results did not demonstrate an increase in the risk of colonisation with or infection with fluconazole-resistant fungi within the time frame of the clinical trials (mean = 29 months, median = 30 months, range = 12–60 months). Whether prophylactic fluconazole will select or induce emergence of fluconazole-resistant fungi in the longer term is still controversial and remains a major consideration before it can be recommended [35-37].

### Limitations of the study

**Figure 6:**
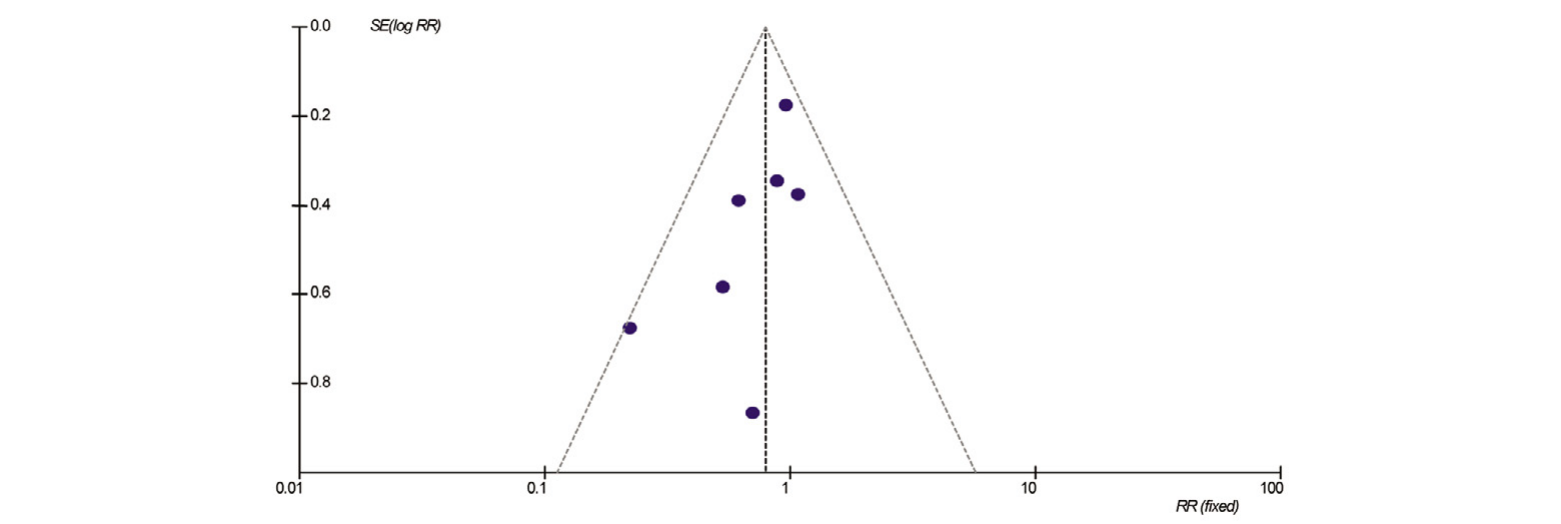
Funnel plot showing the possibility of a small publication bias. RR, relative risk.

Meta-analyses are prone to bias. The quality of trials can affect the direction and magnitude of the treatment effect in meta-analyses. After excluding one study with unclear allocation concealment or double blinding, the direction and magnitude of the results of this meta-analysis remained unchanged. A funnel plot (Figure 6) showed that there was a possibility of a small publication bias, with a lack of small studies showing no effect on mortality with the use of prophylactic fluconazole.

Second, although the results of this meta-analysis were fairly consistent across the included studies, there were significant differences in the diagnoses of the patients and the study protocols, especially in the doses of fluconazole used. The optimal dose and route of administration of fluconazole as a prophylactic agent cannot be evaluated from these pooled studies [32,38].

## Conclusion

In immunocompetent high-risk surgical patients, the use of prophylactic fluconazole is associated with a reduced incidence of candidaemia but with only a trend towards reduction in hospital mortality. A large randomised controlled trial would be needed to assess the cost-effectiveness and the risk of inducing fluconazole-resistant fungi before prophylactic fluconazole can be recommended in immunocompetent high-risk surgical patients.

## Key messages

• 	Prophylactic fluconazole was effective in reducing candidaemia in high-risk surgical patients and was associated with a trend towards a reduction in hospital mortality.

• 	Prophylactic fluconazole was not associated with a significant increase in adverse events or the proportion of patients colonised with or infected with fluconazole-resistant fungi within the time frame of the included studies.

## Abbreviations

CI = confidence interval; ICU = intensive care unit; RR = relative risk

## Competing interests

No financial support was received for this study from pharmaceutical companies or other private companies in the form of grants and awards. All authors declare that they have no competing interests in relation to this study.

## Authors' contributions

KMH performed the data collection and analyses, and drafted the manuscript. JL and GJD helped with the interpretation of data and drafting the manuscript. SARW performed the data collection, and helped with the interpretation of data and drafting the manuscript.

## Figures and Tables

**Table 1:** Characteristics of the included studies

Study	Design	Participants	Interventions	Outcomes	Allocation concealment
Garbino *et al*. [15]	Double-blind	220 patients, mixture of surgical and medical critically ill adult patients; mean age = 54 years, mean APACHE II score = 19.4, mean ICU stay = 8.4 days, mean mortality = 39.5%	Intravenous fluconazole 100 mg/day until a fungal infection developed, withdrawn from mechanical ventilation, or suspicion of a serious adverse event. Duration of study = 30 months	Hospital mortality, proportion of patients with candidaemia, other fungal infections, adverse events requiring cessation of study drug, and patients required rescue therapy using systemic amphotericin B	Adequate
Pelz *et al*. [16]	Double-blind	260 critically ill surgical adult patients; mean age = 64 years, mean APACHE III score = 64, mean ICU stay = 5 days, mean mortality = 11.5%	Loading dose of 800 mg enteral fluconazole followed by 400 mg daily (reduced to 200 mg daily if creatinine clearance <25 ml/min) until 3 days after ICU discharge, or death, or clinical decision to start systemic antifungal therapy. Duration of study = 12 months	Hospital mortality, proportion of patients with candidaemia, and fungal infections, proportion of patients requiring rescue therapy using amphotericin B, proportion of patients colonised with or infected with fluconazole-resistant fungi, and the total length of hospital stay	Adequate
Eggimann *et al*. [17]	Double-blind	43 surgical adult patients with recurrent gut perforation or anastomotic leakage; mean age = 63 years, mean APACHE II score = 13, mean hospital mortality = 39.5%	400 mg intravenous fluconazole daily until complete resolution of the intra-abdominal disease, or development of a fungal infection requiring antifungal therapy, or adverse event related to the study drug. Duration of study = 30 months	Hospital mortality, proportion of patients with candidaemia and other fungal infection, proportion of patients with adverse events leading to cessation of study drug, proportion of patients colonised with or infected with fluconazole-resistant fungi, and the total length of hospital stay	Adequate
Sandven *et al*. [18]	Double-blind	109 high-risk surgical patients with a confirmed intra-abdominal perforation; mean age = 64 years, mean hospital mortality = 11.3%	A single dose of intravenous fluconazole 400 mg intraoperatively. Duration of study 15 months	Hospital mortality, and proportion of patients with fungal infection	Adequate
He *et al*. [19]	Unclear	45 adult patients with severe pancreatitis with at least one organ dysfunction or hyperglycaemia; mean age = 50 years, mean hospital mortality = 20%	Intravenous fluconazole 100 mg/day until no organ failure was observed. Duration of study = 60 months	Hospital mortality, proportion of patients with fungal infection, proportion of patients requiring rescue therapy using amphotericin B, and total length of hospital stay	Unclear
Jacobs *et al*. [13]	Double-blind	34 patients with septic shock from intra-abdominal sepsis; mean age = 50 years, mean APACHE II score = 18, mean ICU stay = 20 days, mean hospital mortality = 44%	Intravenous fluconazole 200 mg/day until resolution of septic shock. Duration of study = 30 months	Hospital mortality, proportion of patients with candidaemia, other fungal infection, proportion of patients requiring rescue therapy using amphotericin B, and proportion of patients colonised with or infected with fluconazole-resistant fungi	Adequate
Ables *et al*. [20]	Double-blind	119 trauma or after intra-abdominal or intra-thoracic surgery adult patients with at least one of the following: central venous catheter, total parenteral nutrition, mechanical ventilation >24 hours, or treatment with broad-spectrum antibiotics; mean age = 44 years, mean APACHE II score = 18, mean mortality = 19.3%	Either Intravenous, oral, or enteral fluconazole 800 mg loading following by 400 mg daily (doses adjusted with renal impairment); oral or enteral route was used when there was a presence of bowel sounds and no history of malabsorption. Duration of study = 26 months	Hospital mortality, proportion of patients with candidaemia, proportion of patients with adverse events leading to cessation of the study drug, proportion of patients colonised with or infected with fluconazole-resistant fungi, and total length of hospital stay	Adequate

APACHE acute physiology and chronic health evaluation; ICU, intensive care unit.
